# Selective autophagy inhibition alleviates oxidative stress-associated neurodegeneration in an AD+CCH model

**DOI:** 10.3389/fnagi.2026.1870441

**Published:** 2026-09-30

**Authors:** Xiaorui Huang, Jufang Chen, Zhicheng Lu, Jie Wang, Xuelian Luo, Qiuyan Qin, Qin Yang, Chongdong Jian, Xuebin Li, Jingwei Shang

**Affiliations:** 1 The First Clinical Medical College of Jinan University, Guangzhou, Guangdong, China; 2 Engineering Research Center of Guangxi Higher Education Institutions for Precise Genetic Testing of Long-dwelling Nationalities, Baise, Guangxi, China; 3 Department of Psychiatry and Psychology, Affiliated Hospital of Youjiang Medical University for Nationalities, Baise, Guangxi, China; 4 Department of Neurology, Baise People's Hospital, Baise, Guangxi, China; 5 Graduate School of Youjiang Medical University for Nationalities, Baise, Guangxi, China; 6 Department of Nephrology, Affiliated Hospital of Youjiang Medical University for Nationalities, Baise, Guangxi, China; 7 Department of Neurology, Affiliated Hospital of Youjiang Medical University for Nationalities, Baise, Guangxi, China; 8 Life Science and Clinical Medicine Research Center, Affiliated Hospital of Youjiang Medical University for Nationalities, Baise, Guangxi, China

**Keywords:** Alzheimer disease, autophagy regulation, chronic cerebral hypoperfusion, oxidative stress response, p62-Keap1-Nrf2 pathway

## Abstract

Dysregulated autophagy is increasingly implicated in the progression of Alzheimer's disease (AD), particularly when chronic cerebral hypoperfusion (CCH) coexists and exacerbates neurodegeneration. However, the relationship between autophagy dysregulation and oxidative stress responses under combined AD+CCH conditions remains incompletely understood. Here, APP/PS1 transgenic mice subjected to CCH were used to evaluate the effects of autophagy modulation with 3-methyladenine (3MA) and rapamycin (RAPA). CCH worsened cognitive impairment and neuronal injury in APP/PS1 mice and was accompanied by altered LC3-II and p62 expression, consistent with disturbed autophagy-related processing. These changes occurred together with increased expression of proteins associated with oxidative stress responses, including Keap1, Nrf2, HO-1, HIF-1α, and NOX-1. Notably, 3MA improved behavioral performance, reduced Aβ40 burden, and was associated with partial normalization of p62, Keap1, Nrf2, and downstream oxidative stress-related proteins. In contrast, RAPA did not confer detectable protection in this model. Age-stratified analyses further showed that abnormalities in autophagy- and oxidative stress-related markers were more severe at 12 months than at 9 months. Collectively, these findings support an association between altered autophagy-related processing and oxidative stress signaling in AD with chronic hypoperfusion and nominate the p62-Keap1-Nrf2 pathway as a candidate mechanism for further causal investigation.

## Introduction

Alzheimer's disease (AD) is the most prevalent neurodegenerative disorder in aging populations and is characterized by progressive memory impairment, cognitive decline, synaptic dysfunction, and neuropsychiatric disturbances. Its complex etiology involves extracellular β-amyloid (Aβ) deposition, intraneuronal tau hyperphosphorylation, chronic neuroinflammation, oxidative stress, and disruption of the neurovascular unit (NVU) (Trombetta et al., 2024; Roemer-Cassiano et al., 2025). With the rapid global demographic shift toward an aging society, AD has become a critical public health challenge, imposing a substantial burden on patients, caregivers, and healthcare systems worldwide (Zhang et al., 2024).

Chronic cerebral hypoperfusion (CCH), defined as a sustained reduction in cerebral blood flow, has emerged as a key contributor to vascular cognitive impairment and a potent accelerator of AD pathology (Zhao and Gong, 2015; Duncombe et al., 2017). CCH-induced microvascular remodeling, blood-brain barrier (BBB) disruption, and metabolic dysregulation foster Aβ overproduction, tau pathology, and neuronal loss in both clinical and animal studies (Zhao and Gong, 2015; Struys et al., 2017). Despite increasing recognition of its pathological significance, the molecular mechanisms by which CCH exacerbates AD remain incompletely understood, particularly with respect to the interplay between oxidative stress and macroautophagy. Notably, how autophagy-oxidative stress crosstalk operates under the combined pathological burden of AD and CCH has not been systematically addressed, representing a critical gap in current knowledge.

Autophagy is a lysosome-dependent degradative process essential for maintaining neuronal proteostasis through the clearance of misfolded proteins, damaged organelles, and aggregated Aβ (Ji et al., 2025). Impairment of autophagy-related processing, especially at the stage of autophagosome-lysosome fusion, has been consistently reported in AD models, where Aβ accumulation itself disrupts autophagy and thereby amplifies neuronal injury (Xu et al., 2024; Palmer et al., 2025). Accordingly, both autophagy activation and inhibition have been proposed as potential therapeutic strategies, depending on disease stage and cellular context. However, the differential consequences of these opposing interventions under CCH-associated metabolic stress remain poorly defined.

Oxidative stress is another central driver of AD pathology. Excessive production of reactive oxygen species (ROS) leads to lipid peroxidation, protein nitration, mitochondrial dysfunction, and oxidative DNA damage (Ma et al., 2024). Furthermore, oxidative stress can promote pathological processes such as neuronal senescence, which has been implicated in AD progression, for instance, through mechanisms involving ceramide accumulation and p53 activation (Wu et al., 2024). The Kelch-like ECH-associated protein 1 (Keap1)-nuclear factor erythroid 2-related factor 2 (Nrf2)-heme oxygenase-1 (HO-1) signaling axis constitutes a primary cellular defense against oxidative injury. Under oxidative conditions, modification of Keap1 allows Nrf2 to translocate into the nucleus and activate antioxidant response element (ARE)-dependent gene expression, including HO-1, thereby restoring redox homeostasis (Fan et al., 2021; Wang et al., 2024). Although both AD and CCH independently induce oxidative imbalance, how this antioxidant pathway is regulated under their combined pathological stress remains unclear. The Nrf2/HO-1 axis orchestrates antioxidant defense while concurrently modulating autophagic and ER-stress pathways in AD, positioning it as a nodal therapeutic target (Wang et al., 2026).

Accumulating evidence indicates a bidirectional regulatory relationship between autophagy and Nrf2 signaling. Autophagic degradation of p62-Keap1 complexes facilitates Nrf2 activation, whereas Nrf2 transcriptionally upregulates multiple autophagy-related genes, together forming an integrated antioxidative-autophagic feedback loop (Picca et al., 2023; Kim et al., 2024; Jo et al., 2014). Disruption of this regulatory network under CCH conditions may therefore exacerbate both oxidative damage and autophagic dysfunction, thereby accelerating AD progression. However, how impaired autophagy-related processing modulates p62-Keap1-Nrf2 signaling in AD under conditions of CCH has not been systematically examined. In the present study, we employed APP/PS1 transgenic mice with or without CCH to investigate the effects of autophagy modulation using rapamycin and 3-methyladenine on oxidative stress and neurodegeneration. By focusing on the p62-Keap1-Nrf2 axis, We hypothesized that AD combined with CCH would produce more pronounced alterations in autophagy-related processing and oxidative stress signaling and that pharmacological modulation of autophagy would differentially affect these pathological and molecular changes.

## Materials and methods

### Experimental animals

This study utilized male C57BL/6J wild-type mice and APP/PS1 double-transgenic mice, aged 30 ± 1 days (mean ± SD). Only male animals were included to avoid confounding effects associated with estrogen-mediated variability (Luo et al., 2022). The APP/PS1 transgenic model expresses two key genes related to AD, enabling the development of robust and heterogeneous AD-like pathology, and is widely recognized for recapitulating early behavioral and neuropathological features of AD (Bilkei-Gorzo, 2014). All animals were housed under specific-pathogen-free (SPF) conditions at the Animal Research Center of Youjiang Medical University for Nationalities. Standard environmental parameters were maintained, including controlled temperature (22 ± 2 °C), humidity (55 ± 5%), and a 12-h light/dark cycle, with free access to water and standard rodent chow. All animal procedures were approved by the Institutional Animal Care and Use Committee (IACUC) of Youjiang Medical University for Nationalities (Approval no. YYFY-LL-2024-265) on March 15, 2024, following the Guide for the Care and Use of Laboratory Animals (8th edition, National Research Council, 2011) and institutional animal welfare guidelines. The study also complied with the ARRIVE 2.0 (Animal Research: Reporting of *In vivo* Experiments) guidelines to ensure transparency in the design, execution, and reporting of *in vivo* animal research. Group assignment was performed by an investigator not involved in behavioral scoring using a random-number table. Behavioral videos (OFT, T-maze, NOR) were scored by an observer blinded to group allocation. The animal cohort used in the present study partially overlaps with that reported in our previous publication; however, the present study addresses a distinct scientific question and reports predominantly new datasets focused on oxidative stress signaling.

### Experimental design and CCH mouse model

For this study, mice were randomly assigned to five experimental groups (Figure 1a): the WT group (C57BL/6J, *n* = 10), the AD group (APP/PS1, *n* = 10), the AD+CCH group (APP/PS1+CCH, *n* = 10), the 3MA group (APP/PS1+CCH+3MA autophagy inhibitor, *n* = 10), and the RAPA group (AD+CCH+RAPA autophagy enhancer, *n* = 10). The CCH model was established using bilateral common carotid artery (BCCA) stenosis with ameroid constrictors (ACs), as previously described in our studies (Yang Q. et al., 2025; Shang et al., 2016, 2019). Briefly, at 4 months of age, mice were anesthetized with a gas mixture of nitrous oxide/oxygen (69%/30%, vol/vol) containing 1% isoflurane and placed in a supine position on a temperature-controlled heating pad. Following midline cervical incision, both common carotid arteries were carefully exposed, and ameroid constrictors were positioned around each artery. To confirm successful induction of chronic cerebral hypoperfusion, cerebral blood flow (CBF) was monitored using Laser Doppler Flowmetry under isoflurane anesthesia. Baseline CBF was recorded before AC placement, followed by measurements immediately after surgery and on postoperative days 1, 3, 7, 14, and 28. Only animals exhibiting a stable reduction in CBF to approximately 60–70% of baseline values were included in subsequent analyzes. Progressive narrowing of the BCCAs resulted in a sustained decrease in CBF, reaching 60–70% of baseline by postoperative day 28.

**Figure 1:**
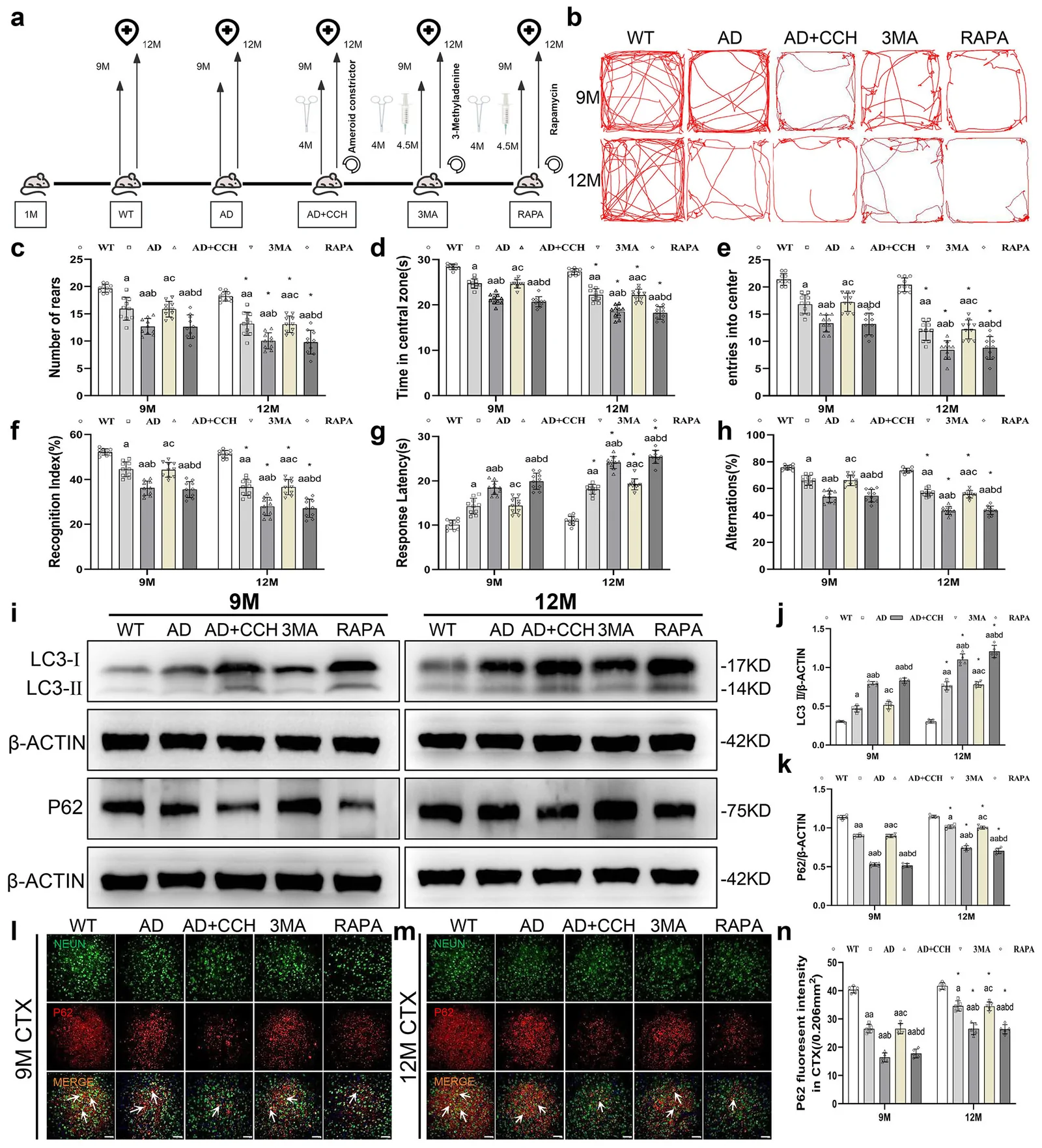
Schematic of experimental design and timeline. 5-month-old male WT (C57BL/6J) and APP/PS1 mice were randomized into five groups (a, *n* = 10/group): WT, AD (APP/PS1), AD+CCH (APP/PS1 + bilateral common carotid artery stenosis with ameroid constrictors, AC), AD+CCH+3MA, and AD+CCH+RAPA. AC denotes the ameroid constrictor placed around each common carotid artery to induce chronic cerebral hypoperfusion (CCH); the ring symbol on the carotid diagram indicates AC placement. **(b–e)** Open-field test for 9-month-old and 12-month-old mice, assessing rearing frequency, time spent in the central zone, and entries into the central area. **(f)** Novel object recognition (NOR) test evaluating recognition memory, expressed as recognition index (RI). **(g, h)** T-maze assessing spatial working memory, including latency to correct arm entry and percentage of correct choices. **(i)** Representative immunoblots showing LC3-II and p62 protein expression in different mouse groups. Quantification of LC3-II **(j)** and p62 **(k)** expression levels. Representative images of double immunofluorescence staining showing co-localization of NeuN (green) and p62 (red) in cortical neurons of 9-month-old **(l)** and 12-month-old **(m)** mice (arrowheads). **(n)** Quantitative analysis of P62 intensity in the CTX. (*n* = 5, a: *p* < 0.05 vs. WT, aa: *p* < 0.01 vs. WT; b: *p* < 0.05 vs. AD; c: *p* < 0.05 vs. AD+CCH; d: *p* < 0.05 vs. 3MA; *: *p* < 0.05 for comparison within the same group between 12 and 9 months. Scale bar: 50 μm. M, month; WT, wild-type; AD, Alzheimer's disease; CCH, chronic cerebral hypoperfusion; 3MA, 3-methyladenine; RAPA, rapamycin; CTX, cortex.)

### Autophagy modulator treatment protocol

Beginning on postoperative day 15 after the induction of CCH, mice assigned to the intervention arms received intraperitoneal injections of autophagy modulators. Specifically, 3MA (30 mg/kg, Sigma-Aldrich) and RAPA (6 mg/kg, CST, 9904S) were administered twice weekly and continued until the animals were sacrificed. For preparation, 3-MA was dissolved in sterile saline, whereas RAPA was diluted in a vehicle consisting of 10% dimethyl sulfoxide (DMSO) and 90% saline. Drug administration was initiated on postoperative day 15, corresponding to the completion of progressive arterial constriction induced by ameroid constrictors. At this time point, cerebral blood flow is stabilized at a hypoperfused level, allowing pharmacological interventions to be applied during an established rather than evolving CCH state. These dosing regimens were carried over from our previously established protocol in the same APP/PS1 ± CCH cohort (Yang Q. et al., 2025). 3-MA (30 mg/kg, ip, biweekly) is a canonical Vps34/Class III PI3K inhibitor used to block autophagosome initiation in AD models. Mechanistically, 3-MA suppresses Vps34-dependent autophagosome formation and prevents dysfunctional lysosomal storage in neuronal models (Zhang X. et al., 2021). RAPA (6 mg/kg, ip, biweekly) inhibits mTORC1. Intraperitoneal RAPA administration in APP/PS1 transgenic mice has been shown to inhibit mTOR, promote autophagosome-lysosome degradation, reduce senile plaque deposition, and ameliorate AD pathology (Chen et al., 2019). Importantly, recent commentary notes that while RAPA reduces AD neuropathological hallmarks when administered early, in later-stage AD—where the lysosomal system is severely damaged—RAPA treatment may actually exacerbate lysosomal damage, rendering it a “double-edged sword”(Carosi and Sargeant, 2019). 3-MA targets initiation; RAPA targets mTORC1-dependent flux promotion. Under conditions where lysosomal clearance is already compromised (aged AD+CCH), promoting autophagosome formation without restoring degradation may be deleterious, whereas suppressing excessive initiation (3-MA) may be comparatively beneficial.

### Behavioral assessment

Behavioral testing was performed to evaluate locomotor activity, anxiety-related behavior, and cognitive function. The open field test (OFT) was used to assess general locomotion and anxiety-like behavior, thereby controlling for potential confounding effects on cognitive performance. The novel object recognition (NOR) test was employed to assess recognition memory, which is sensitive to hippocampal and cortical dysfunction. The T-maze test was used to evaluate spatial working memory dependent on prefrontal-hippocampal circuitry. Together, these assays provide complementary measures of behavioral domains relevant to AD and CCH pathology.

### Open field test

The OFT was conducted in mice aged 9 and 12 months across all experimental groups. Each mouse was individually placed in the center of a square polycarbonate arena (120 cm × 120 cm), with a predefined central zone measuring 100 cm × 100 cm. Prior to testing, animals were acclimated to the behavioral testing room for 60 min. Locomotor activity was recorded for 10 min using an automated video-tracking system (Smart 2.5; Panlab, Barcelona, Spain). Total distance traveled, average velocity, time spent in the central zone, and movement trajectories within the central zone were quantified. After each trial, the apparatus was cleaned with 75% ethanol to eliminate residual olfactory cues.

### T-maze test

The T-maze apparatus consisted of one start arm and two identical goal arms arranged in a T-shaped configuration. Mice were initially allowed to freely explore the maze to facilitate habituation. During training trials, a food reward was placed at the distal end of one goal arm. Following repeated training, spatial working memory was assessed in a probe trial in which the reward was removed. The arm selected, and the latency to arm entry were recorded as indices of spatial working memory performance.

### Novel object recognition test

The NOR test was conducted in an open-field arena. During the familiarization phase, two identical objects were placed symmetrically in the arena, each positioned 15 cm from the walls and separated by 25 cm. Mice were allowed to explore freely for 10 min. After a 5-h intertrial interval, one familiar object was replaced with a novel object of similar size but different shape and color. Animals were then reintroduced to the arena for a 10-min test session. Exploration time directed toward each object was recorded using an overhead video-tracking system. Recognition memory was quantified using the recognition index (RI), calculated as follows: RI = [time spent exploring the novel object / (time spent exploring the novel object + familiar object)] × 100%.

### Brain tissue processing

At 9 and 12 months of age, mice were deeply anesthetized with pentobarbital sodium (40 mg/kg, intraperitoneal injection) and transcardially perfused with 20 mL of ice-cold phosphate-buffered saline (PBS). Brains were rapidly removed and either snap-frozen at −80 °C for biochemical analyzes or further processed for histological assessment. For histology, animals were additionally perfused with 20 mL of 4% paraformaldehyde (PFA, 0.1 M in PBS). Brains were post-fixed in 4% PFA overnight at 4 °C, dehydrated, cleared, embedded in paraffin, and sectioned for subsequent staining.

### Western blot analysis

Following PBS perfusion, the cerebral cortex (CTX) and hippocampus (HI) were dissected separately and homogenized on ice in lysis buffer, as these regions exhibit early vulnerability to both AD pathology and CCH. The homogenates were centrifuged at 12,000 rpm for 15 min at 4 °C, and the supernatants were collected for protein extraction. Five animals were included in each experimental group (*n* = 5). Total protein concentrations were quantified using the bicinchoninic acid (BCA) assay. Equal amounts of protein (20 μg per sample) were denatured in loading buffer and separated via sodium dodecyl sulfate-polyacrylamide gel electrophoresis (SDS-PAGE). The resolved proteins were then transferred onto polyvinylidene fluoride (PVDF) membranes (MERCK) using the wet transfer technique. Membranes were blocked for 1 h at ambient temperature with 5% non-fat milk diluted in Tris-buffered saline containing 0.1% Tween-20 (TBST) to minimize non-specific interactions. Subsequently, membranes were incubated overnight at 4 °C with primary antibodies specific to target proteins. After washing with TBST, membranes were incubated with horseradish peroxidase (HRP)-conjugated secondary antibodies for 1 h at room temperature. Protein detection was performed using enhanced chemiluminescence (ECL) reagents, and band intensities were quantified with ImageJ software. For each protein, 3 technical replicates per biological sample (*n* = 5 mice/group) were averaged. Data are presented as mean ± SEM with individual data points overlaid (scatter-plot format).

The primary antibodies used included: LC3I/II (1:1000, 14600-1-AP, Proteintech), p62/SQSTM1 (1:1000, 380612, Zenbio), Nrf2 (1:1000, 380773, Zenbio), Keap1 (1:1000, A17062, ABclonal), APP (1:1000, R23330, Zenbio), HO-1 (1:1000, 10701-1-AP, Proteintech), NQO-1 (1:1000, 11451-1-AP, Proteintech), SOD-1 (1:1000, R27324, Zenbio), HIF-1α (1:1000, 340462, Zenbio), NOX-1 (1:1000, 17772-1-AP, Proteintech), HSP70 (1:1000, 10995-1-AP, Proteintech), MRP-1 (1:1000, PB0977, Boster), and β-actin (1:5000, 66009-1-Ig, Proteintech) as an internal loading control. Immunoreactive bands were visualized using a Bio-Rad ChemiDoc MP system. To avoid signal oversaturation, exposure durations were optimized using automated settings, and multiple exposures were acquired to select those within the linear dynamic range of detection. This ensured quantitative fidelity of protein expression measurements. Western blot analysis was performed on cerebral cortex (CTX) homogenates for Figures 1i, 3a, 4a, 5a.

### Immunofluorescence assessment

Tissue sections embedded in paraffin were first deparaffinized, followed by incubation in 3% hydrogen peroxide for 15 min to quench endogenous peroxidase activity. After thorough rinsing with PBS, antigen retrieval was performed using 0.1 M sodium citrate buffer. Non-specific binding was blocked using normal serum for 1 h at room temperature. Subsequently, primary antibodies derived from rabbit and mouse sources were co-incubated with the sections overnight at 4 °C. The next day, following PBS washes, fluorescently conjugated secondary antibodies were applied for 1 h at room temperature. DAPI 4′, 6-diamidino-2-phenylindole) was used for nuclear counterstaining with a 10-min incubation. After a final PBS wash, fluorescence imaging was conducted using a laser scanning confocal microscope equipped with argon and HeNe1 lasers (LSM-510; Zeiss, Jena, Germany). Paraffin sections were imaged using a Zeiss LSM-510 confocal microscope. Within each staining batch, laser power, detector gain, offset, pinhole size, and scanning settings were kept identical across groups. Secondary-only controls (no primary antibody) were run in parallel for each antibody batch to exclude cross-reactivity artifacts. Representative images were selected from raw fields that were free of tissue folds, edge vignetting, and overt sectioning artifacts. Quantification was performed on raw, unprocessed images in ImageJ using an identical analysis pipeline across groups after standardized background subtraction. For each animal, three non-overlapping sections and predefined regions of interest were analyzed.

The antibodies employed for immunofluorescence included: anti-amyloid β40 (1:200, 014-26923, Wako), SQSTM1/p62 (1:1000, 380612, Zenbio), Nrf2 (1:1000, 380773, Zenbio), Keap1 (1:1000, A17062, ABclonal), HO-1 (1:1000, 10701-1-AP, Proteintech), NQO-1 (1:1000, 11451-1-AP, Proteintech), SOD-1 (1:1000, R27324, Zenbio), HIF-1α (1:1000, 340462, Zenbio), NOX-1 (1:1000, 17772-1-AP, Proteintech), HSP70 (1:1000, 10995-1-AP, Proteintech), and NeuN (1:200, ab177487, Abcam; 1:200, 66836-1-Ig, Proteintech). Secondary antibodies included Goat anti-Mouse IgG (H+L), highly cross-adsorbed (1:500, A-11001, Invitrogen), and Donkey anti-Rabbit IgG (H+L), highly cross-adsorbed (1:500, A-21207, Invitrogen). Immunofluorescence staining intensity for Aβ40, Keap1 and NRF2 in the cortex (CTX) and hippocampus (HI), as well as P62, HO-1, NQO-1, SOD-1, HIF-1α, NOX-1, and HSP-70 in the motor cortex, specifically in layer 4, was quantified using ImageJ software (National Institutes of Health, Bethesda, MD, USA). For each brain, three separate sections were analyzed. Representative images were captured under consistent exposure settings to ensure comparability. The image intensities were measured, and regions of interest (ROIs) were defined to include the stained areas. Background fluorescence was subtracted from each ROI to obtain accurate measurements.

### Statistical analysis

Statistical analyses were performed using SPSS 23.0 and GraphPad Prism 10.0. Data are presented as mean ± SD. For endpoints measured in both 9-month-old and 12-month-old mice, a two-way ANOVA was used with group (WT, AD, AD+CCH, AD+CCH+3MA, AD+CCH+RAPA) and age (9 vs. 12 months) as between-subject factors, followed by Tukey's multiple-comparison test when appropriate. Because the experimental design was not fully factorial for AD background, CCH, and drug treatment, biologically prespecified *post hoc* contrasts were emphasized: WT vs. AD, AD vs. AD+CCH, AD+CCH vs. AD+CCH+3MA, and AD+CCH vs. AD+CCH+RAPA. For variables assessed within a single age stratum, one-way ANOVA followed by Tukey's multiple-comparison test was used. Data distribution and homogeneity of variance were assessed using the Shapiro-Wilk and Levene tests, respectively. When assumptions were violated, data were transformed or analyzed using Kruskal-Wallis testing followed by Dunn's multiple-comparison test, as appropriate. Outliers in behavioral datasets were screened using the Grubbs test (alpha = 0.05) before formal analysis.

## Results

### Assessment of cognitive impairment

Cognitive functions were evaluated among the experimental groups using a battery of behavioral paradigms, including the open-field test, T-maze, and NOR task (Figures 1b–h). In the open-field assay (Figures 1b–e), two-way ANOVA showed significant main effects of group and age on all measured parameters. For rearing activity, there were significant main effects of group [*F*(4, 40) = 45.44, *p* = 0.00012] and age [*F*(1, 20) = 35.35, *p* = 0.00016], with a significant group × age interaction [*F*(4, 40) = 0.3824, *p* = 0.0199]. *Post hoc* analysis confirmed that rearing activity was significantly reduced in AD mice vs. WT (adjusted *p* = 0.0252) and was further reduced in AD+CCH mice compared with AD mice (adjusted *p* = 0.0168). Administration of the autophagy inhibitor 3MA partially reversed this deficit vs. the AD+CCH group (adjusted *p* = 0.0299), whereas treatment with RAPA did not (adjusted *p* = 0.9988). Similarly, for time spent in the central area, two-way ANOVA revealed significant main effects of group [*F*(4, 40) = 58.81, *p* = 0.00012] and age [*F*(1, 20) = 34.56, *p* = 0.00014], with no significant interaction [*F*(4, 40) = 0.7839, *p* = 0.5424]. *Post hoc* tests indicated this measure was reduced in AD vs. WT (adjusted *p* = 0.00045) and further reduced in AD+CCH vs. AD (adjusted *p* = 0.0052). Treatment with 3MA partially reversed this deficit (adjusted *p* = 0.0071 vs. AD+CCH), whereas RAPA did not (adjusted *p* = 0.9870). For the frequency of entries into the central zone, two-way ANOVA showed significant main effects of group [*F*(4, 40) = 86.67, *p* = 0.00011] and age [*F*(1, 20) = 29.39, *p* = 0.0006], as well as a significant interaction [*F*(4, 40) = 3.443, *p* = 0.0189]. *Post hoc* analysis demonstrated a reduction in AD vs. WT (adjusted *p* = 0.0045), a further reduction in AD+CCH vs. AD (adjusted *p* = 0.0156), and a partial reversal by 3MA (adjusted *p* = 0.0155 vs. AD+CCH), but not by RAPA (adjusted *p* = 0.9955). No significant between-group differences in rearing activity, central zone time, or central zone entry frequency were observed, indicating that locomotor activity and baseline anxiety do not confound the cognitive assessments from T-maze and NOR.

In the NOR test (Figure 1f), two-way ANOVA revealed significant effects of group [*F*(4, 40) = 72.89, *p* = 0.00013] and age [*F*(1, 20) = 34.76, *p* = 0.0004] on the recognition index, with a significant interaction [*F*(4, 40) = 2.714, *p* = 0.0472]. *Post hoc* analysis showed that the recognition index was reduced in AD mice vs. WT (adjusted *p* = 0.006) and further reduced in AD+CCH mice vs. AD mice (adjusted *p* = 0.0023). 3MA partially reversed this deficit vs. AD+CCH (adjusted *p* = 0.003), whereas RAPA did not differ from AD+CCH (adjusted *p* = 0.9973). Results from the T-maze (Figures 1g, h) for latency showed significant effects of group [*F*(4, 40) = 88.86, *p* = 0.00014] and age [*F*(1, 20) = 75.25, *p* = 0.0003], with a significant interaction [*F*(4, 40) = 3.806, *p* = 0.0122]. *Post hoc* analysis indicated latency was increased in AD mice vs. WT (adjusted *p* = 0.0073) and further increased in AD+CCH mice vs. AD mice (adjusted *p* = 0.0077). 3MA partially reversed this increase (adjusted *p* = 0.0101 vs. AD+CCH), whereas RAPA did not (adjusted *p* = 0.6356). For the percentage of correct choices, two-way ANOVA revealed significant effects of group [*F*(4, 40) = 66.32, *p* = 0.00011] and age [*F*(1, 20) = 107.1, *p* = 0.00018], with no significant interaction [*F*(4, 40) = 2.130, *p* = 0.0999]. *Post hoc* analysis confirmed that correct choice percentages were reduced in AD vs. WT (adjusted *p* = 0.0078) and further reduced in AD+CCH vs. AD (adjusted *p* = 0.0007). 3MA treatment partially restored performance (adjusted *p* = 0.006 vs. AD+CCH), while RAPA did not (adjusted *p* = 0.9329). In addition, an age-related decline was observed, as all groups except the WT cohort showed further deterioration at 12 months relative to 9 months in latency, recognition performance, and decision-making accuracy.

### Autophagic activity alterations analysis

To evaluate changes in autophagic activity across experimental groups, the expression levels of LC3-II and p62 were assessed by Western blotting (Figures 1i–k). At both 9 and 12 months of age, the AD and AD+CCH groups exhibited changes consistent with altered autophagic processing, as indicated by significantly increased LC3-II levels (Figures 1i, j; *p* < 0.05, *p* < 0.01) and markedly decreased p62 expression (Figures 1i, k; *p* < 0.05, *p* < 0.01). Notably, the reduction in p62 expression was more pronounced in the AD+CCH group compared to the AD group alone (*p* < 0.05). Of note, the concurrent reduction in p62 alongside LC3-II elevation precludes a straightforward “flux-blocked” interpretation and may reflect enhanced p62 turnover as an autophagy substrate, or a stage-dependent dysregulation wherein autophagosome formation is upregulated but substrate fate is altered (see Discussion). In the absence of direct flux assays (CQ/BafA1 chase or mRFP-GFP-LC3), we report these changes as “altered autophagic processing” rather than definitive flux impairment. Pharmacological intervention further confirmed these observations. Treatment with 3MA partially corrected the autophagic abnormalities in AD+CCH mice, resulting in a significant decrease in LC3-II and a concomitant restoration of p62 levels compared to untreated controls (*p* < 0.05). In contrast, treatment with RAPA did not produce measurable changes in LC3-II or p62 expression. In addition, age-dependent effects on autophagy were observed. Compared to 9-month-old mice, those at 12 months showed greater changes in LC3-II and p62 levels across experimental groups (Figures 1i–k; *p* < 0.05). These biochemical findings were further supported by immunofluorescence co-localization of p62 and the neuronal marker NeuN in cortical tissue, which visually demonstrated changes in p62 expression within neurons (Figures 1l–n).

Two-way ANOVA revealed significant effects of group and age on both markers. For LC3-II, there were significant effects of group [*F*(4, 40) = 241.9, *p* = 0.00019] and age [*F*(1, 20) = 44.70, *p* = 0.0026], with a significant interaction [*F*(4, 40) = 14.64, *p* = 0.00011]. *Post hoc* analysis showed that LC3-II levels were increased in AD mice vs. WT (adjusted *p* = 0.0059) and further increased in AD+CCH mice vs. AD mice (adjusted *p* = 0.0004). Treatment with 3MA significantly decreased LC3-II vs. the AD+CCH group (adjusted *p* = 0.0018), whereas RAPA had no effect (adjusted *p* = 0.0813).

### Expression changes of upstream proteins in the Keap1-Nrf2-HO-1 signaling pathway

Western blot analysis (Figures 2a–d) revealed significant alterations in the expression levels of key upstream proteins involved in the Keap1-Nrf2-HO-1 signaling pathway, including APP, Keap1 and Nrf2 (*p* < 0.05, *p* < 0.01). Compared with the WT group, both the AD and AD+CCH groups exhibited markedly elevated expressions of these proteins, with the AD+CCH group showing a more pronounced increase (*p* < 0.05). Following the pharmacological intervention, treatment with 3MA significantly downregulated the expression of APP, Keap1, and Nrf2 in the brains of AD+CCH mice (Figures 2b–d; *p* < 0.05). In contrast, RAPA treatment did not produce measurable changes in the expression of these proteins. 3-MA downregulated Keap1 and Nrf2 expression (*p* < 0.05), indicating partial normalization of the oxidative stress response.

**Figure 2:**
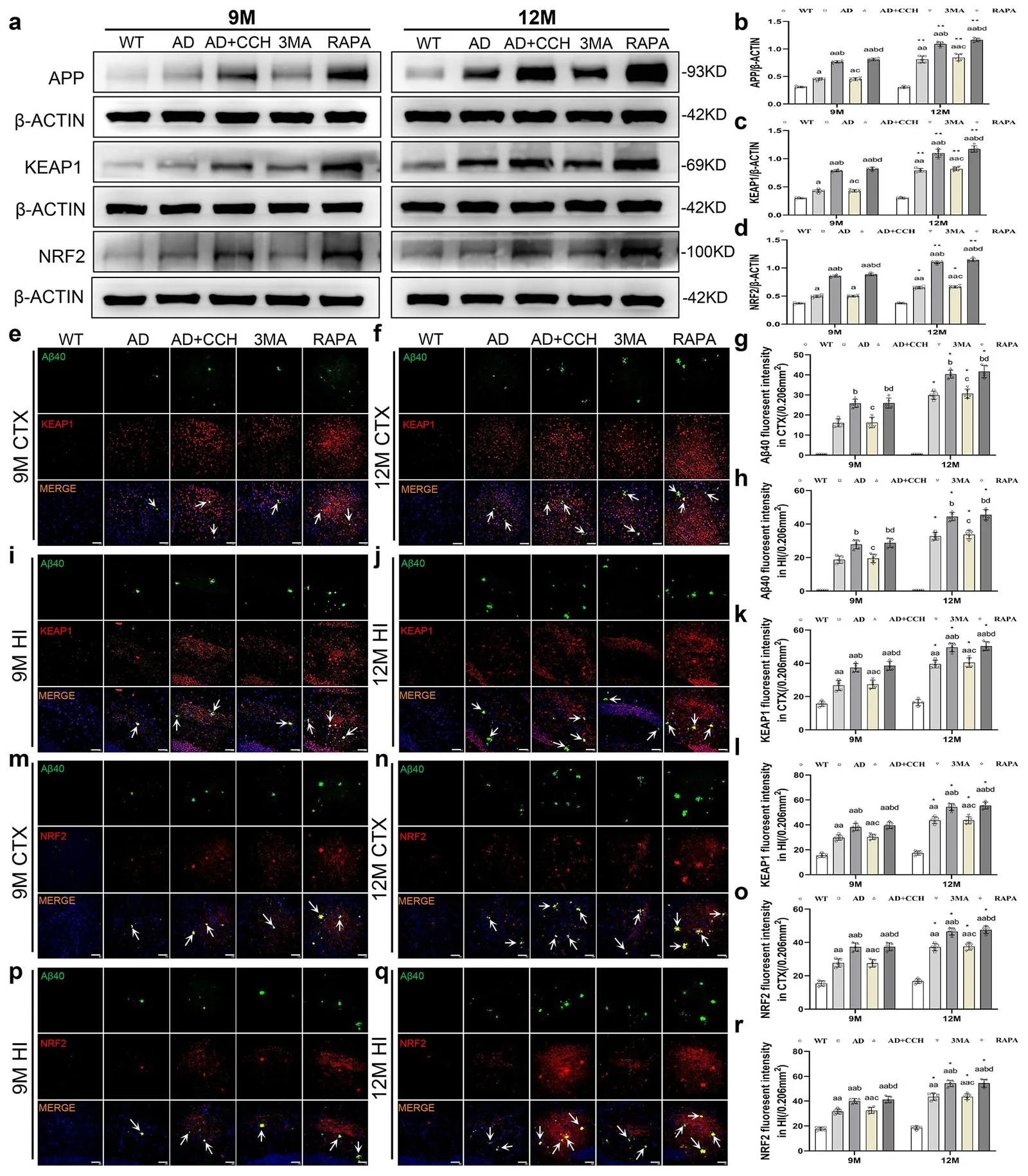
Analysis of upstream protein expression in the Keap1-Nrf2-HO-1 signaling pathway and Aβ40 deposition. **(a)** Representative Western blot images showing the expression levels of APP, Keap1 and Nrf2 in different mouse groups. Quantification of APP **(b)**, Keap1 **(c)** and Nrf2 **(d)** levels. Representative images of double immunofluorescence staining showing co-localization of Aβ40 (green) and Keap1 (red) in the CTX **(e, f)** and HI **(i, j)** of 9- and 12-month-old mice (arrowheads). Quantitative analysis of Aβ40 **(g, h)** and Keap1 **(k, l)** intensity in the CTX and HI. Double immunofluorescence staining of Aβ40 (green) and Nrf2 (red) in CTX **(m, n)** and HI **(p, q)**, with merged images showing co-localization (arrowheads). Quantitative analysis of Nrf2 intensity in the CTX **(o)** and HI **(r)**. (*n* = 5, a: *p* < 0.05 vs. WT, aa: *p* < 0.01 vs. WT; b: *p* < 0.05 vs. AD; c: *p* < 0.05 vs. AD+CCH; d: *p* < 0.05 vs. 3MA; *: *p* < 0.05 for comparison within the same group between 12 and 9 months. Scale bar: 50 μm. M, month; WT, wild-type; AD, Alzheimer's disease; CCH, chronic cerebral hypoperfusion; 3MA, 3-methyladenine; RAPA, rapamycin; CTX, cortex; HI, hippocampus).

Further age-related analysis showed that in the AD, AD+CCH, and intervention groups, 12-month-old mic e displayed significantly higher levels of APP, Keap1, and Nrf2 compared to 9-month-old mice, showing an age-related increase in the expression of these proteins (Figures 2b–d; *p* < 0.05). Immunofluorescence double-labeling further confirmed these findings. In both the cerebral cortex (CTX, Figures 2e, f, m, n) and hippocampus (HI, Figures 2i, j, p, q), co-localization of Keap1 and Nrf2 with Aβ40 was markedly enhanced in the AD and AD+CCH groups (Figures 2k, l, o, r). Notably, aberrant accumulation of Aβ40 was observed in the CTX and HI of AD mice, which was further exacerbated by CCH (Figures 2g, h).

Two-way ANOVA also showed significant main effects of group and age for all three proteins. For APP, there were significant effects of group [*F*(4, 40) = 741.2, *p* = 0.00018] and age [*F*(1, 20) = 165.1, *p* = 0.0002], with a significant interaction [*F*(4, 40) = 76.79, *p* = 0.0001]. *Post hoc* tests indicated APP was increased in AD vs. WT (adjusted *p* = 0.0021) and further increased in AD+CCH vs. AD (adjusted *p* = 0.00014). 3MA downregulated APP in AD+CCH mice (adjusted *p* = 0.0001), whereas RAPA did not (adjusted *p* = 0.3833). For Keap1, two-way ANOVA revealed significant effects of group [*F*(4, 40) = 707.1, *p* = 0.00011] and age [*F*(1, 20) = 244.9, *p* = 0.00019], with a significant interaction [*F*(4, 40) = 54.45, *p* = 0.00012]. Levels were increased in AD vs. WT (adjusted *p* = 0.0043) and further in AD+CCH vs. AD (adjusted *p* = 0.00015). 3MA reduced Keap1 (adjusted *p* = 0.0001 vs. AD+CCH), but RAPA did not (adjusted *p* = 0.8391). For Nrf2, two-way ANOVA revealed significant effects of group [*F*(4, 40) = 624.7, *p* = 0.0004] and age [*F*(1, 20) = 180.4, *p* = 0.0002], with a significant interaction [*F*(4, 40) = 21.45, *p* = 0.00013]. Expression was increased in AD vs. WT (adjusted *p* = 0.0022) and further in AD+CCH vs. AD (adjusted *p* = 0.0006). 3MA downregulated Nrf2 (adjusted *p* = 0.00021 vs. AD+CCH), while RAPA had no effect (adjusted *p* = 0.7013).

### Alterations in downstream protein expression of the Keap1-Nrf2-HO-1 signaling pathway

Western blot analysis (Figure 3a) revealed a significant upregulation of downstream antioxidant enzymes HO-1, NQO-1, and SOD-1 in the brain tissues of AD mice compared with the WT group (Figures 3b–d; *p* < 0.05, *p* < 0.01), reflecting changes associated with the Keap1-Nrf2 signaling cascade. Notably, the expression levels of these proteins were further elevated in the AD+CCH group compared to the AD group alone (*p* < 0.05), showing higher levels under CCH conditions. In pharmacological intervention experiments, treatment with 3MA significantly reduced the expression of HO-1, NQO-1, and SOD-1 in the AD+CCH group (*p* < 0.05). In contrast, RAPA treatment did not produce measurable changes in the expression of these proteins. Age-dependent analysis further demonstrated that 12-month-old mice exhibited markedly higher levels of HO-1, NQO-1, and SOD-1 than 9-month-old mice, displaying a clear trend of age-related upregulation (Figures 3a–d; *p* < 0.05).

**Figure 3:**
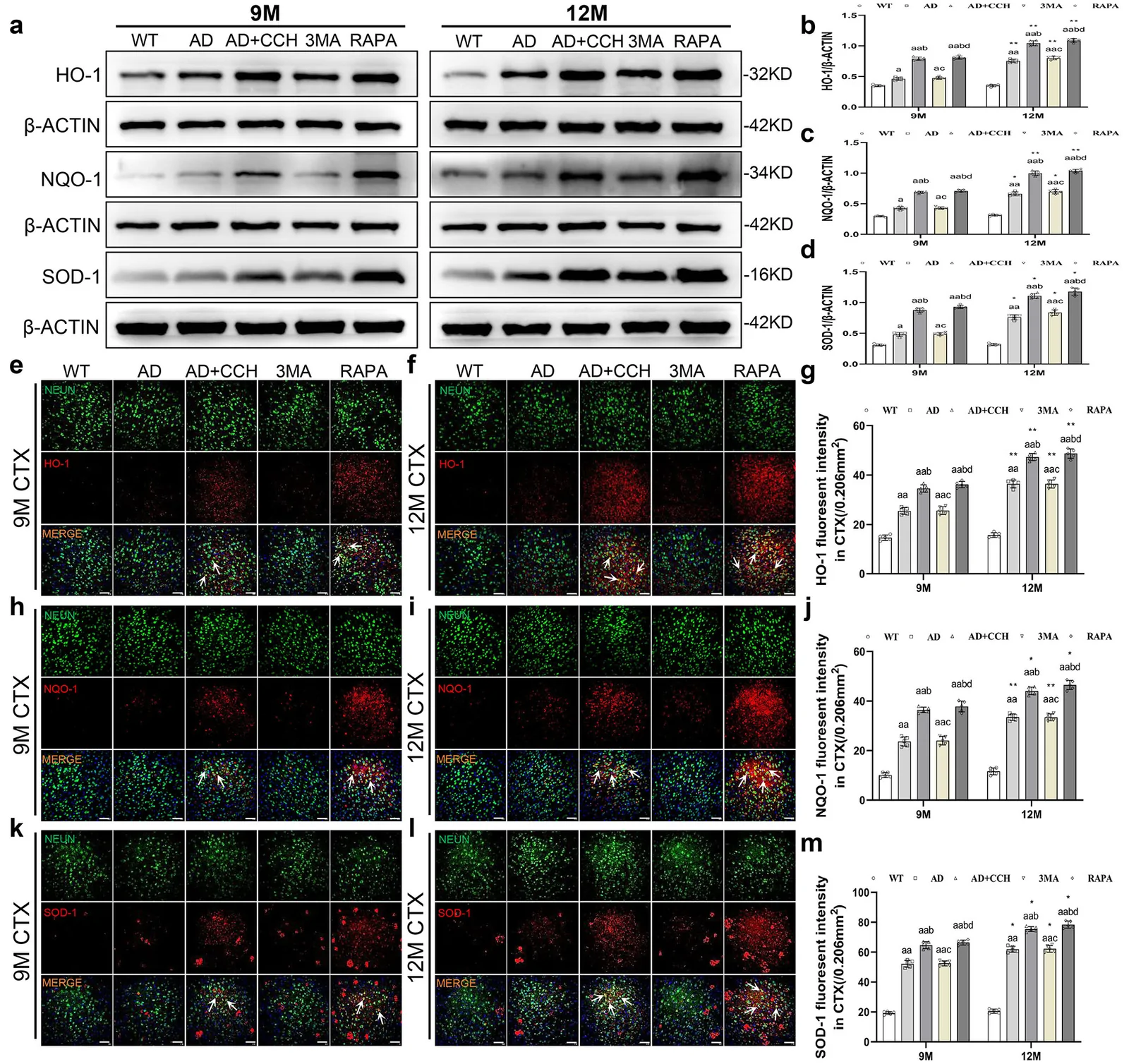
Altered expression of downstream antioxidant enzymes in the Keap1-Nrf2-HO-1 signaling pathway. **(a)** Representative Western blot images showing the expression levels of HO-1, NQO-1 and SOD-1 in different mouse groups. Quantification of HO-1**(b)**, NQO-1 **(c)** and SOD-1 **(d)** levels. Double immunofluorescent staining of NeuN (green) and HO-1 red; **(e, f)**, NeuN (green) and NQO-1 red; **(h, i)**, NeuN (green) and SOD-1 red; **(k, l)** in CTX for 9-month-old mice and 12-month-old mice, merged images highlighting neuronal co-localization of antioxidant enzymes (arrowheads). Quantitative analysis of HO-1 **(g)**, NQO-1 **(j)** and SOD-1 **(m)** intensity in the CTX. (*n* = 5, a: *p* < 0.05 vs. WT, aa: *p* < 0.01 vs. WT; b: *p* < 0.05 vs. AD; c: *p* < 0.05 vs. AD+CCH; d: *p* < 0.05 vs. 3MA; *: *p* < 0.05 for comparison within the same group between 12 and 9 months. Scale bar: 50 μm. M, month; WT, wild-type; AD, Alzheimer's disease; CCH, chronic cerebral hypoperfusion; 3MA, 3-methyladenine; RAPA, rapamycin; CTX, cortex).

For HO-1, there were significant effects of group [*F*(4, 40) = 874.0, *p* = 0.00018] and age [*F*(1, 20) = 143.0, *p* = 0.0003], with a significant interaction [*F*(4, 40) = 63.38, *p* = 0.00041]. Levels were increased in AD vs. WT (adjusted *p* = 0.0016) and further in AD+CCH vs. AD (adjusted *p* = 0.00021). 3MA reduced HO-1 (adjusted *p* = 0.00015 vs. AD+CCH), but RAPA did not (adjusted *p* = 0.9110). For NQO-1, two-way ANOVA revealed significant effects of group [*F*(4, 40) = 789.7, *p* = 0.00016] and age [*F*(1, 20) = 80.64, *p* = 0.0009], with a significant interaction [*F*(4, 40) = 52.52, *p* = 0.00032]. Expression was increased in AD vs. WT (adjusted *p* = 0.0013) and further in AD+CCH vs. AD (adjusted *p* = 0.0022). 3MA decreased it (adjusted *p* = 0.0003 vs. AD+CCH), whereas RAPA did not (adjusted *p* = 0.3112). For SOD-1, two-way ANOVA revealed significant effects of group [*F*(4, 40) = 575.6, *p* = 0.00035] and age [*F*(1, 20) = 77.74, *p* = 0.0008], with a significant interaction [*F*(4, 40) = 26.01, *p* = 0.00024]. Levels were increased in AD vs. WT (adjusted *p* = 0.0014) and further in AD+CCH vs. AD (adjusted *p* = 0.0037). 3MA reduced SOD-1 (adjusted *p* = 0.0025 vs. AD+CCH), while RAPA had no effect (adjusted *p* = 0.2061).

Immunofluorescence staining visually demonstrated these expression patterns (Figures 3e, f, h, i, k, l). In the CTX region, HO-1, NQO-1, and SOD-1 co-localized with NeuN, with the most pronounced expression observed in the AD and AD+CCH groups (Figures 3e–m).

### Expression changes of oxidative stress-related proteins

Western blot analysis (Figure 4a) revealed that the expression levels of oxidative stress-related proteins HIF-1α, NOX-1, HSP-70, and MRP-1 which were significantly upregulated in the brains of both AD and AD+CCH groups compared with the WT group (Figures 4b–e; *p* < 0.05, *p* < 0.01). Notably, the AD+CCH group exhibited a more pronounced increase than the AD group alone (*p* < 0.05). Following pharmacological intervention, 3MA treatment significantly reduced the expression of HIF-1α, NOX-1, HSP-70, and MRP-1 in the AD+CCH group (*p* < 0.05), while RAPA treatment did not result in measurable changes. The age-stratified analysis further demonstrated that 12-month-old mice in all experimental groups exhibited markedly higher expression levels of the aforementioned proteins compared to their 9-month-old counterparts (Figures 4a–e; *p* < 0.05), showing an age-related increase in these protein levels.

**Figure 4:**
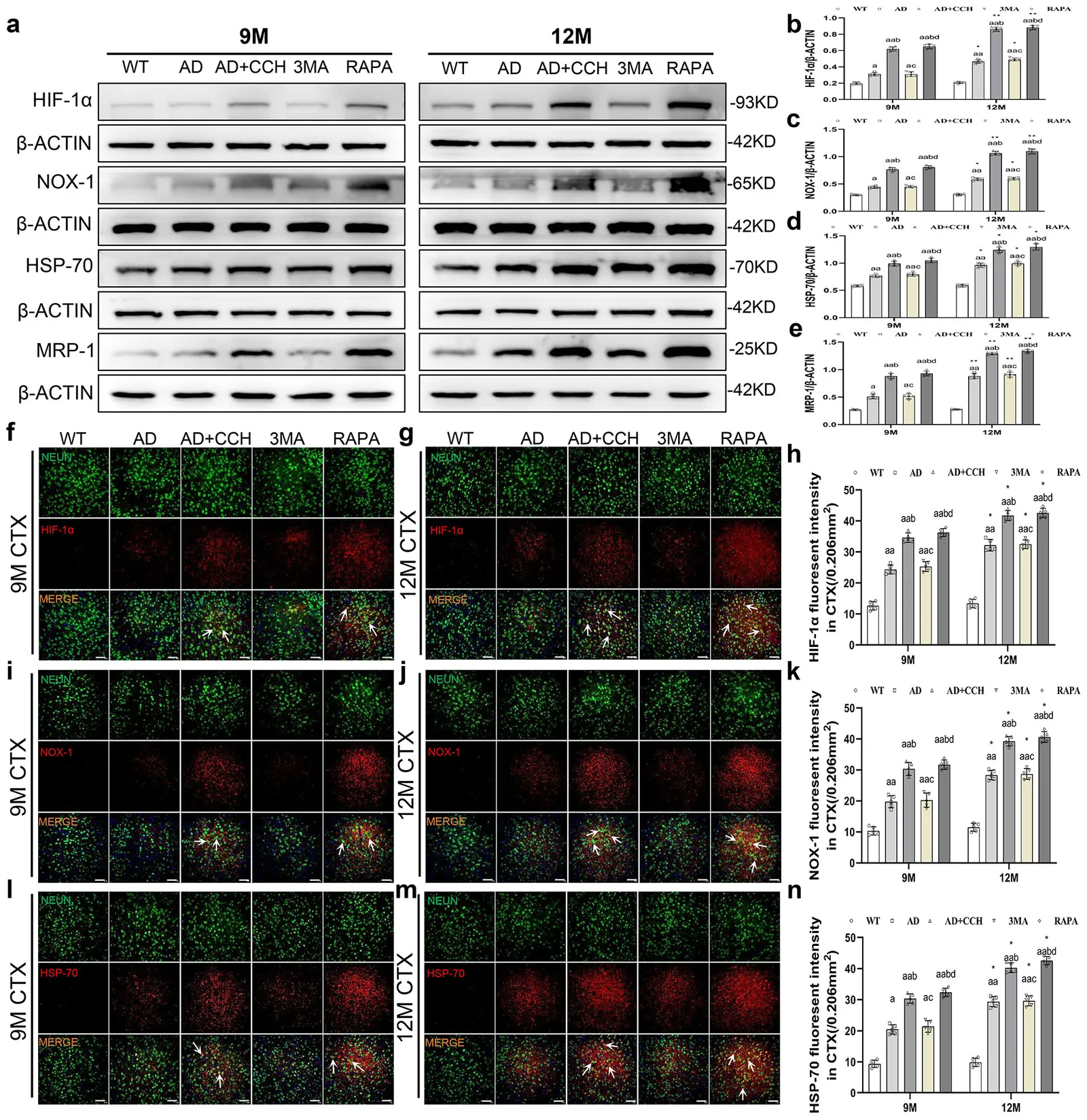
Assessment of oxidative stress-related proteins in mouse brains. **(a)** Representative Western blot images showing the expression levels of HIF-1α, NOX-1, HSP-70, and MRP-1 in different experimental groups. Quantitative analysis of HIF-1α **(b)**, NOX-1 **(c)**, HSP-70 **(d)**, and MRP-1 **(e)** protein levels. Representative double immunofluorescence images showing co-localization of NeuN (green) with HIF-1α red; **(f, g**), NOX-1 red; **(i, j**), and HSP-70 red; **(l, m**) in the CTX of 9- and 12-month-old mice (arrowheads). **(n)** Quantitative analysis of HIF-1α **(h)**, NOX-1 **(k)** and HSP-70 **(n)** intensity in the CTX. (*n* = 5, a: *p* < 0.05 vs. WT, aa: *p* < 0.01 vs. WT; b: *p* < 0.05 vs. AD; c: *p* < 0.05 vs. AD+CCH; d: *p* < 0.05 vs. 3MA; *: *p* < 0.05 for comparison within the same group between 12 and 9 months. Scale bar: 50 μm. M, month; WT, wild-type; AD, Alzheimer's disease; CCH, chronic cerebral hypoperfusion; 3MA, 3-methyladenine; RAPA, rapamycin; CTX, cortex).

For HIF-1α, two-way ANOVA revealed significant effects of group [*F*(4, 40) = 857.2, *p* = 0.00051] and age [*F*(1, 20) = 120.3, *p* = 0.0004], with a significant interaction [*F*(4, 40) = 30.97, *p* = 0.00014]. Expression was increased in AD vs. WT (adjusted *p* = 0.0025) and further in AD+CCH vs. AD (adjusted *p* = 0.00028). 3MA reduced it (adjusted *p* = 0.00033 vs. AD+CCH), but RAPA did not (adjusted *p* = 0.6070). For NOX-1, two-way ANOVA revealed significant effects of group [*F*(4, 40) = 467.4, *p* = 0.00028] and age [*F*(1, 20) = 209.9, *p* = 0.0001], with a significant interaction [*F*(4, 40) = 20.49, *p* = 0.00017]. Levels were increased in AD vs. WT (adjusted *p* = 0.0015) and further in AD+CCH vs. AD (adjusted *p* = 0.0045). 3MA decreased NOX-1 (adjusted *p* = 0.00034 vs. AD+CCH), whereas RAPA did not (adjusted *p* = 0.7096). For HSP70, two-way ANOVA revealed significant effects of group [*F*(4, 40) = 224.3, *p* = 0.00022] and age [*F*(1, 20) = 34.95, *p* = 0.0041], with a significant interaction [*F*(4, 40) = 10.59, *p* = 0.0002]. Expression was increased in AD vs. WT (adjusted *p* = 0.0036) and further in AD+CCH vs. AD (adjusted *p* = 0.0008). 3MA reduced it (adjusted *p* = 0.0024 vs. AD+CCH), while RAPA had no effect (adjusted *p* = 0.5996).

Immunofluorescence co-labeling visually demonstrated expression patterns consistent with the Western blot findings (Figures 4f, g, i, j, l, m). In the CTX, HIF-1α, NOX-1, HSP-70, and MRP-1 were co-localized with NeuN, with markedly enhanced signals observed in both the AD and AD+CCH groups (Figures 4f–n).

### 3 -MA attenuated Keap1 and Nrf2 immunoreactivity in the cortex and hippocampus of AD+CCH mice

To corroborate the biochemical findings at the tissue level, immunofluorescence staining was performed to examine Keap1 and Nrf2 expression in the CTX and HI of 9− and 12-month-old mice (Figure 5a-d). In the CTX, Keap1 immunoreactivity was progressively elevated from WT to AD and further to AD+CCH mice at both ages (*p* < 0.05 for all intergroup comparisons), and this increase was significantly curtailed by 3-MA (*p* < 0.05 vs. AD+CCH), whereas RAPA exerted no detectable effect (Figure 5a, e). Cortical Nrf2 immunoreactivity followed an identical pattern, with the AD+CCH group exhibiting the highest signal, which was markedly attenuated by 3-MA (*p* < 0.01 vs. AD+CCH) but not by RAPA (Figure 5a, g). Comparable regional changes were observed in the hippocampus for both Keap1 and Nrf2, with significant increases in AD and AD+CCH mice versus WT controls (*p* < 0.05 for Keap1, *p* < 0.01 for Nrf2), further elevations in AD+CCH versus AD (*p* < 0.05 for both), and robust reductions following 3-MA treatment (*p* < 0.05 for Keap1, *p* < 0.01 for Nrf2), while RAPA again failed to produce significant alterations (Figure 5c, d, f, h). Across all regions and markers, immunoreactivity was more pronounced at 12 months than at 9 months within the AD, AD+CCH and 3MA groups (*p* < 0.05), indicating an age-dependent aggravation.

**Figure 5:**
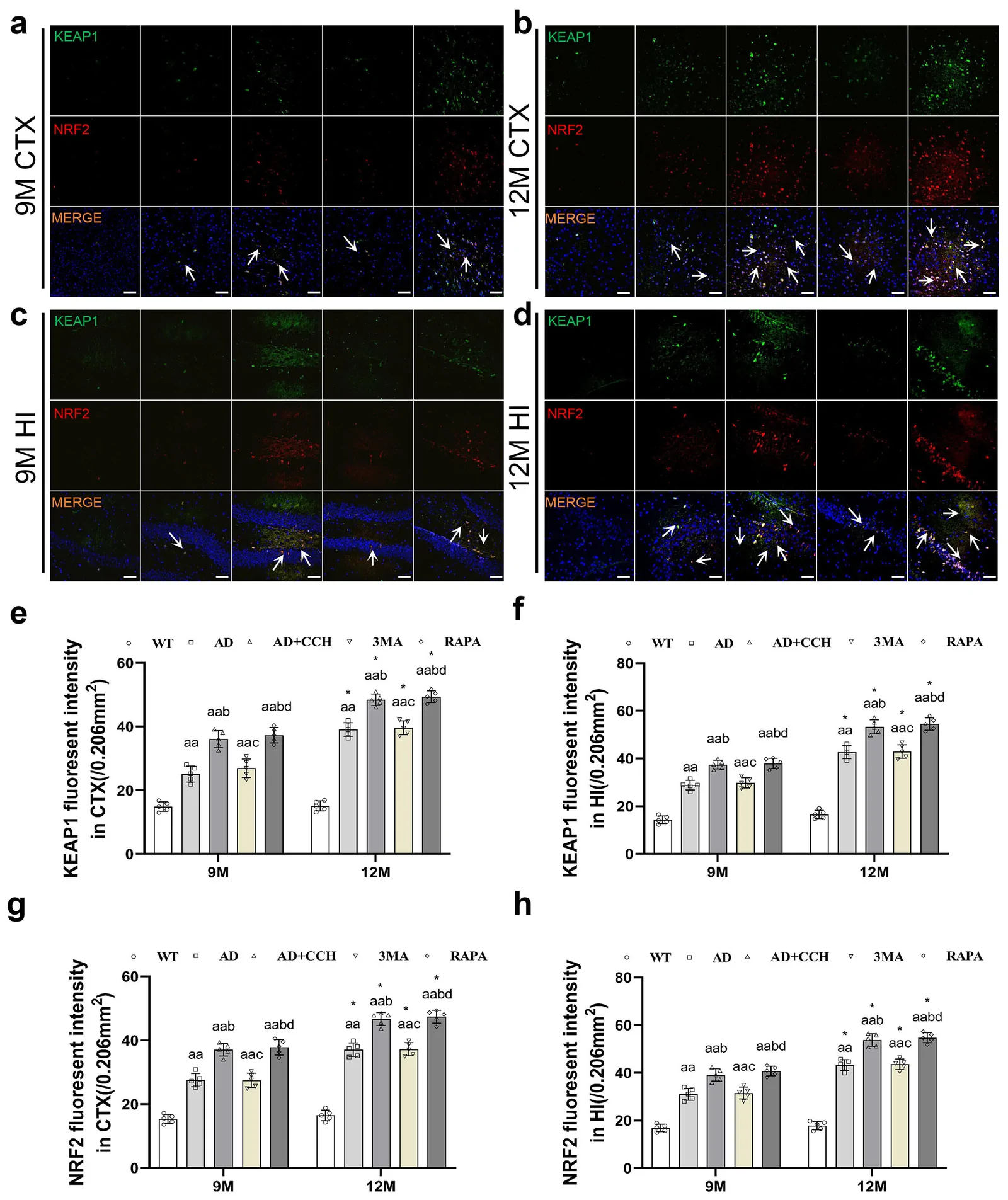
3-MA attenuates Keap1 and Nrf2 immunoreactivity in the cortex and hippocampus of AD+CCH mice. Representative immunofluorescence images showing Keap1 (green) and Nrf2 (red) staining in the CTX **(a, b)** and HI **(c, d)** of 9− and 12-month-old mice from each experimental group, with merged images demonstrating regional expression patterns. Quantitative analyses of Keap1 **(e, f)** and Nrf2 **(g, h)** fluorescence intensity in the CTX **(e, g)** and HI **(f, h)**. (n = 5, a: *p* < 0.05 vs WT, aa: *p* < 0.01 vs WT; b: *p* < 0.05 vs AD; c: *p* < 0.05 vs AD+CCH; d: *p* < 0.05 vs 3MA; ^*^: *p* < 0.05 for comparison within the same group between 12 and 9 months. Scale bar: 50 μm. M: month; WT: wild-type; AD: Alzheimer's disease; CCH: chronic cerebral hypoperfusion; 3MA: 3-methyladenine; RAPA: rapamycin; CTX: cortex.)

## Discussion

CCH has been implicated in AD progression through disruption of neurovascular unit integrity, amplification of neuroinflammation, and impairment of mitochondrial homeostasis (Xiao et al., 2024). Recent evidence further suggests that ischemic/hypoxic stress can promote Arginase-1-dependent mitochondrial cristae remodeling and PANoptosis (She et al., 2025). In the present study, CCH aggravated cognitive impairment (Figure 1) and Aβ deposition (Figure 2) in APP/PS1 mice, accompanied by alterations in autophagy-related processing (Figure 1) and oxidative stress-responsive proteins (Figure 4). Changes in Keap1-Nrf2-HO-1-related proteins (Figures 2–5) were observed in parallel at the biochemical and tissue levels, whereas 3-MA was associated with improvement in behavioral and pathological outcomes and partial normalization of these molecular changes (Figures 1–6). These findings indicate a close association between altered autophagy and oxidative stress responses in AD under chronic hypoperfusion, but do not establish a causal sequence between these processes.

**Figure 6:**
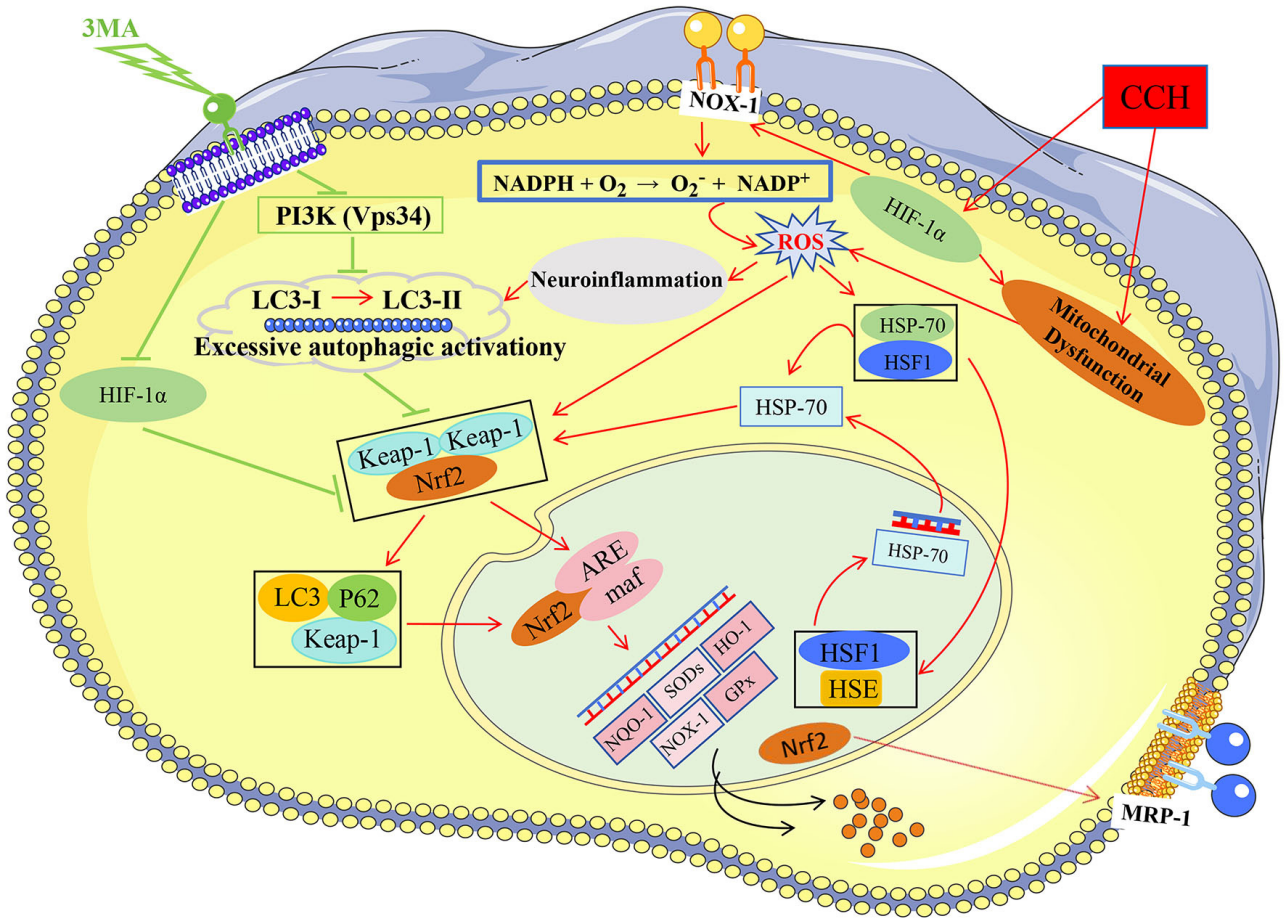
Adaptive antioxidant response modulated by autophagy inhibition in AD+CCH. This schematic illustrates the molecular mechanisms through which inhibition of autophagy modulates the adaptive antioxidant response under conditions of AD complicated by CCH. CCH induces mitochondrial dysfunction and activates NOX-1, leading to excessive production of ROS. ROS contributes to neuroinflammation and upregulation of HIF-1α and HSP-70 via activation of HSF1. Concurrently, ROS promotes excessive autophagic activation, characterized by the conversion of LC3-I to LC3-II, which is mediated by PI3K (Vps34). The autophagy inhibitor 3MA suppresses this pathway.

CCH has been recognized as a major factor precipitating early cognitive deterioration (Rajeev et al., 2023). In the present study, a systematic behavioral assessment across various murine groups demonstrated that CCH exposure markedly intensified cognitive dysfunction in AD models, with age-dependent worsening (Figure 1). Correspondingly, changes in autophagy-related protein expression were observed in groups exposed to CCH, in parallel with the behavioral impairments. Notably, previous studies have suggested that excessive autophagy may contribute to hippocampal neuronal vulnerability and cognitive decline (Di Meco et al., 2020). Pharmacological intervention with 3MA led to notable improvements in behavioral outcomes, which is consistent with the interpretation that autophagy dysregulation is involved in the neurocognitive deficits observed under CCH conditions. These findings are consistent with earlier research emphasizing the neuroprotective potential of autophagy suppression in pathological contexts such as cerebral ischemia (Chen et al., 2014).

CCH has been shown to influence autophagy-related processes in brain tissue and has been associated with the pathogenesis of neurodegenerative diseases (Xu et al., 2023). In this study, a combined AD+CCH mouse model was employed to investigate the modulatory effects of CCH on autophagy in AD. Analysis of key autophagy markers revealed significantly increased LC3-II levels in both AD and AD+CCH groups, with the most substantial elevation observed in the combined model (Figure 1). This was accompanied by a reduction in p62 expression, indicating that CCH is associated with more pronounced changes in autophagy-related protein expression in the AD brain, potentially in a time-dependent manner. Previous studies have proposed that CCH may influence autophagy through pathways involving neuroinflammation and lysosomal function (Su et al., 2017). Convergent 2025 evidence from APP/PS1 models indicates that pharmacological modulation of the p62/Keap1/Nrf2 complex concomitantly alters LC3-II, Beclin1, Atg7, Atg12, SOD, and CAT (Zhang et al., 2025), while CCH models demonstrate that excessive mitophagy drives cognitive impairment and its suppression recapitulates neuroprotection (Li et al., 2025).

Autophagic processing in the AD+CCH brain: interpreting LC3-II and p62. While concurrent elevation of both LC3-II and p62 is a classic indicator of impaired autophagy-related processing (i.e., blocked degradation at the autophagosome-lysosome fusion stage), our finding of reduced p62 amidst elevated LC3-II points to a more complex scenario. It may indicate enhanced turnover of p62 itself as an autophagy substrate, or a stage-dependent dysregulation where autophagosome formation is upregulated but the subsequent fate of substrates like p62 is altered through mechanisms potentially involving transcriptional regulation or feedback loops. The more pronounced decrease in p62 in the AD+CCH group further suggests that chronic cerebral hypoperfusion may exacerbate autophagy-related imbalance in the AD brain (Lu et al., 2024). Given that p62 is a selective substrate cleared via autophagy, its accumulation alongside LC3-II has been reported to occur in contexts where autophagosome-lysosome fusion or lysosomal degradation capacity is reduced (Li et al., 2022; Pankiv et al., 2007). Age-related studies in AD transgenic models have described increased senile plaque formation, autophagosome accumulation, and reduced autolysosome formation with advancing age, consistent with age-related alterations in autophagy pathways (Long et al., 2020). These findings support the interpretation that CCH may exacerbate autophagy-related alterations in AD and suggest that therapeutic strategies aimed at modulating autophagy-related processing merit further investigation in the context of CCH-associated AD progression.

Accordingly, in the absence of direct flux confirmation (CQ/BafA1 chase or mRFP-GFP-LC3), the Results section reports LC3-II/p62 changes as “altered autophagic processing” rather than a definitive flux blockade—a qualification that applies throughout the following interpretation. This interpretation is aligned with emerging consensus that NRF2 directly transcriptionally targets autophagy-related genes including SQSTM1/p62 and TFE3 (Chu et al., 2026), providing a mechanistic basis for the p62-centric regulatory node proposed here.

In the AD+CCH model, 3MA significantly suppressed excessive autophagic activation, suggesting a potential therapeutic benefit under this dual pathological condition. Early in AD, autophagy promotes neuroprotection by removing misfolded proteins and maintaining neuronal homeostasis (Uddin et al., 2019). However, disease progression leads to Aβ and hyperphosphorylated tau accumulation, overloading the lysosomal system, impairing autophagy-lysosome function, and reducing pathogenic protein clearance. This dysfunction can initiate autophagy-dependent cell death (Hamano et al., 2018; Nilsson and Saido, 2014). CCH has been reported to influence these processes by increasing metabolic stress, oxidative burden, and neuroinflammatory signaling, which may further contribute to autophagy-related alterations (Kimura et al., 2025). In this context, 3MA effectively attenuated pathological autophagy signaling and reduced neuronal injury, supporting the relevance of modulating autophagy in settings of comorbid neurodegeneration.

The divergent responses to 3-MA vs. RAPA further illustrate this context dependence. 3-MA suppresses excessive autophagosome initiation, thereby reducing p62 accumulation. Conversely, RAPA, an mTOR inhibitor and autophagy inducer, while promoting autophagy-related processing, fails to confer benefit because lysosomal clearance is already compromised—autophagosomes form but cannot mature to autolysosomes. This is consistent with recent CCH studies demonstrating that excessive mitophagy drives cognitive impairment, and its suppression (not induction) is neuroprotective (Li et al., 2025). RAPA's “double-edged sword” effect in late-stage AD with lysosomal damage is increasingly recognized (Carosi and Sargeant, 2019). This contrast between 3-MA (effective) and RAPA (ineffective) is unlikely to reflect an inherent inability of mTOR inhibition to modify AD pathology; rather, it likely reflects disease-stage- and context-dependent constraints on autophagy modulation. Early In AD, autophagy induction may confer neuroprotection provided that autophagy-related processing and lysosomal function remain intact. However, when the degradative arm is compromised, further enhancement of autophagosome formation may promote vacuolar accumulation without facilitating clearance of autophagic cargo. This interpretation aligns with stage-dependence studies showing that rapamycin abolishes cognitive deficits and reduces amyloid-β levels in AD mouse models when administered before substantial plaque deposition, with diminished or absent benefit at advanced disease stages (Spilman et al., 2010). Consistent with this view, prior studies (Chen et al., 2025) in 3xTg-AD mice demonstrated that rapamycin administration improved AD-related pathology only when initiated before the formation of substantial amyloid plaques and neurofibrillary tangles, with no observable benefit thereafter. Thus, the lack of therapeutic effect in our AD+CCH model may reflect the superimposition of chronic cerebral hypoperfusion onto an already compromised autophagic-lysosomal background in an age-vulnerable context, thereby narrowing the window for effective autophagy induction. Collectively, these observations underscore that the biological context of autophagy modulation—particularly disease stage, lysosomal integrity, and the presence of vascular-metabolic stress—must be carefully considered when interpreting the effects of rapamycin in neurodegenerative diseases. Additionally, prior studies have suggested that impairments in autophagosome-lysosome fusion or lysosomal degradation, including altered acidification or enzyme activity, may limit the completion of autophagy-related processing and complicate the interpretation of autophagy markers such as LC3-II, which could contribute to the lack of observable effects with RAPA. These results suggest the importance of considering autophagy flux integrity and disease stage when designing interventions targeting this pathway. Inappropriate activation under conditions of flux impairment may worsen neuronal injury, highlighting the need for stage-specific therapeutic strategies.

Autophagy and oxidative stress exhibit intricate crosstalk, particularly in neurodegenerative disorders, where their interplay critically influences disease progression. This study highlights the involvement of the Keap1-Nrf2-HO-1 axis, a key oxidative stress-responsive pathway in this interaction. The Keap1-Nrf2-HO-1 pathway is a major redox-responsive system that regulates cellular antioxidant defenses. Under oxidative stress, Nrf2 can escape Keap1-mediated degradation, translocate to the nucleus, and induce antioxidant genes, including HO-1, NQO-1, and SOD-1 (Wang et al., 2026). In the present AD+CCH model, the increased abundance of Nrf2 and its downstream antioxidant proteins (Figures 2–5) may reflect a compensatory response to sustained oxidative stress rather than constitutive activation of Nrf2 signaling (Figure 6). Such changes have been associated in prior studies with mitochondrial dysfunction, impaired proteostasis, and alterations in autophagy pathways. Notably, inhibition of autophagy by 3MA suppressed Nrf2 signaling while alleviating cognitive decline and Aβ deposition, suggesting that modulation of autophagy may influence oxidative stress-related signaling and functional outcomes, which is consistent with recent findings on autophagy modulation in neurodegeneration (Deng et al., 2022). p62 provides an important molecular link between autophagy and Nrf2 signaling by binding Keap1 and influencing Nrf2 stability (Zhang W. et al., 2021). However, the direction and functional consequence of this interaction may depend on cellular context, disease stage, and the status of autophagic processing. In the present study, p62 expression changed in parallel with LC3-II and with Keap1-Nrf2-related proteins following 3-MA treatment, but these associations do not establish a causal regulatory sequence. Direct manipulation of p62 or Keap1 will therefore be required to determine whether the p62-Keap1 interaction mediates the observed effects of autophagy modulation. The parallel increases in Keap1 and Nrf2 detected by Western blotting were further supported by immunofluorescence in both the cortex and hippocampus, particularly in 12-month-old AD+CCH mice (Figure 5). Rather than indicating a simple linear relationship between Keap1 abundance and Nrf2 activity, these findings are more consistent with coordinated regulation of the pathway under sustained cellular stress. Nevertheless, the absence of direct measurements of Nrf2 nuclear localization or transcriptional activity limits interpretation of these changes at the functional level. These patterns resemble autophagy-ROS interactions reported in ischemic brain injury (Hou et al., 2022) and suggest that the p62-Keap1-Nrf2 pathway may be a relevant target for future therapeutic investigation in AD with cerebrovascular comorbidities.

The concomitant increase in Keap1 and Nrf2 observed in the AD+CCH groups does not necessarily contradict the established Keap1-Nrf2 regulatory framework. Rather, these changes may reflect an adaptive response to sustained oxidative stress at the level of protein expression, rather than a direct indication of Nrf2 functional activation. Under persistent oxidative stress, increased Nrf2 expression may represent an attempt to reinforce antioxidant defense, while Keap1 expression may also increase as part of the cellular feedback mechanisms that constrain Nrf2 signaling. Thus, increased Keap1 abundance does not necessarily imply enhanced Nrf2 degradation or suppression of Nrf2-dependent responses, particularly when oxidative stress alters the functional interaction between these proteins. In the present study, the parallel reduction of Keap1 and Nrf2 following 3-MA treatment further indicates that these changes were coordinated with the broader stress response. However, because nuclear translocation, DNA-binding activity, and transcriptional activity of Nrf2 were not directly assessed, the present data do not establish Nrf2 activation or define the directionality of Keap1-Nrf2 regulation. This distinction is important when interpreting the Keap1-Nrf2 findings in the context of autophagy-related changes.

CCH has been associated with changes in AD pathology that involve alterations in the HIF-1α/NOX-1 and ER stress (ERS)/MRP-1 pathways. HIF-1α is stabilized under hypoxic conditions, circumventing proteasomal degradation to induce adaptive responses such as angiogenesis and metabolic reprogramming (Zeng et al., 2022). In parallel, NOX-1, a major source of ROS, is upregulated in AD, promoting lipid peroxidation and mitochondrial dysfunction (Vitale et al., 2024). In the AD+CCH model, HIF-1α and NOX-1 expression were further elevated, and 3MA treatment reduced their expression levels (Figure 4), a pattern consistent with the possibility that autophagy modulation may influence HIF-1α and NOX-1-related pathways.

ERS leads to the upregulation of HSP-70, which functions as a molecular chaperone to maintain proteostasis and prevent protein aggregation (Song et al., 2016). Concurrently, MRP-1, an ATP-dependent efflux transporter, facilitates the removal of glutathione-conjugated oxidative metabolites to mitigate cellular toxicity (Xu et al., 2020). Both HSP-70 and MRP-1 were markedly increased in AD and further elevated under CCH, while 3MA administration significantly reduced their levels (Figure 4), a pattern consistent with increased ER stress and oxidative burden under these conditions, which may contribute to elevated HSP-70 and MRP-1 expression. Collectively, these findings are consistent with the interpretation that CCH is associated with concurrent changes in HIF-1α/NOX-1-related ROS signaling and ER stress-associated MRP-1 responses in AD (Figure 6). In this study, autophagy inhibition with 3MA was associated with reduced expression of HIF-1α, NOX-1, HSP-70, and MRP-1, which may reflect broader effects on cellular stress pathways, including mitochondrial and ER-related processes. This pathological interplay is consistent with autophagy-ROS dynamics observed in cerebral ischemia-reperfusion injury models (Zheng et al., 2025). Together with recent reports showing that antioxidant strategies can alleviate AD-related pathological changes (Jia et al., 2024), our findings support the view that correcting redox imbalance—either directly or through upstream pathways such as autophagy—may represent a promising therapeutic approach for AD, particularly in the setting of cerebrovascular insufficiency.

Further investigation will be required to determine how autophagy modulators influence HIF-1α-regulated angiogenic signaling, the substrate specificity of MRP-1, and the interplay among autophagy, oxidative stress, and neuroinflammation in AD complicated by CCH. Emerging evidence on epitranscriptomic regulation, including m6A-dependent control of neuroinflammation (Han et al., 2025), may provide an additional avenue for future mechanistic work in this area. From a translational perspective, our findings do not support indiscriminate enhancement or suppression of autophagy in AD with cerebrovascular insufficiency. Instead, they argue for a stage-specific and flux-aware therapeutic strategy. Thus, the stage-specific and flux-aware framework proposed here converges with recent mechanistic advances in NRF2-autophagy transcriptional coupling (Chu et al., 2026) and Nrf2/HO-1-axis-targeted therapeutic strategies in AD (Huangfu et al., 2025). One potential implication is that the p62-Keap1-Nrf2 node may represent a more selective therapeutic entry point than global manipulation of autophagosome biogenesis. In settings characterized by oxidative overload and incomplete autophagy-related processing, strategies that restore autophagic completion, improve lysosomal competence, or rebalance p62-dependent redox signaling may be more rational than broad mTOR-directed interventions alone. Future translational studies should therefore incorporate biomarkers capable of distinguishing increased autophagosome formation from failed autophagic clearance (Yang N. et al., 2025), particularly in patients with mixed neurodegenerative and vascular pathology.

### Limitations

Several limitations of the present study should be acknowledged. First, only male mice were included, which may limit the generalizability of the findings, as sex-dependent differences in autophagy, oxidative stress responses, and Alzheimer's disease pathology have been reported. Future studies incorporating both sexes will be important to determine whether the observed effects extend across genders.

Second, direct autophagy-related processing assays (CQ/BafA1 chase, mRFP-GFP-LC3) were not performed; thus LC3-II and p62 changes are reported as “altered autophagic processing” rather than definitive flux blockade.

Third, the study relied on pharmacological modulation of autophagy using 3-methyladenine and rapamycin. Although these agents are widely used experimental tools, they may have off-target effects that complicate the interpretation of autophagy-specific mechanisms. Genetic approaches, such as conditional deletion or overexpression of autophagy-related genes, would provide additional mechanistic precision.

Fourth, the analyzes were conducted at only two time points (9 and 12 months), which capture middle and later stages of disease progression. More continuous temporal sampling would help delineate dynamic changes in autophagy-related processing, oxidative stress signaling, and neuropathological development across the full disease trajectory.

Fifth, regarding the Keap1/Nrf2-Aβ40 co-labeling in Figure 2, near-complete overlap raised the possibility of primary-antibody cross-reactivity. We re-stained archival sections with Keap1- and Nrf2-specific primaries in the absence of anti-Aβ40, together with secondary-only controls. The outcome of this validation is reported in the corresponding figure legend, and causality regarding the p62-Keap1-Nrf2 axis remains to be established by p62 knockdown or Keap1 rescue experiments.

### Conclusion

The present study demonstrates that chronic cerebral hypoperfusion aggravates cognitive and pathological abnormalities in APP/PS1 mice and is accompanied by alterations in autophagy-related processing and oxidative stress-responsive signaling. Treatment with 3-MA was associated with improved behavioral and pathological outcomes and partial normalization of several autophagy- and oxidative stress-related proteins, whereas rapamycin produced no comparable detectable benefit. The coordinated changes in p62, Keap1, Nrf2, and downstream proteins are consistent with an interaction between autophagy-related processes and redox signaling; however, the present data do not establish a causal regulatory relationship or demonstrate that the protective effects of 3-MA are mediated through the p62-Keap1-Nrf2 pathway. Further studies incorporating direct autophagy-related processing measurements and genetic manipulation of p62 or Keap1 will be necessary to define the underlying molecular mechanisms.

## Data Availability

The raw data supporting the conclusions of this article will be made available by the authors, without undue reservation.

## Author disclaimer

The animal cohort used in this study (APP/PS1±CCH±3-MA±RAPA, IACUC YYFY-LL-2024-265) was also reported in the authors' previous work: Yang Q. et al., Inflammation2025, 48:541-556 (DOI: 10.1007/s10753-024-02043-0). The 2024 article focused on neuroinflammation (IBA1, GFAP, IL-1β, TNF-α) and included a subset of baseline behavioural (T-maze, NOR) and autophagy-marker (LC3-II, p62 Western blot; p62+NeuN immunofluorescence) data as contextual validation. The present manuscript addresses a distinct hypothesis-autophagy-oxidative stress crosstalk via the p62-Keap1-Nrf2 axis and reports predominantly new data: Keap1/Nrf2/Aβ 40 co-labelling (Figure 2), the full Keap1-Nrf2-HO-1 downstream panel (HO-1, NQO-1, SOD-1; Figure 3) the oxidative-stress panel (HIF-1α, NOX-1, HSP70, MRP-1; Figure 4) and the 3-MA-effective / RAPA-ineffective mechanistic contrast, all absent from the 2024 article. Western-blot sample size was increased from n = 3 to n = 5/group. All data reuse falls within the original IACUC-approved protocol scope.
